# Early Diagnosis in Cerebellar Ataxia, Neuropathy, Vestibular Areflexia Syndrome (CANVAS) by Focusing on Major Clinical Clues: Beyond Ataxia and Vestibular Impairment

**DOI:** 10.3390/biomedicines10082046

**Published:** 2022-08-22

**Authors:** Laurent Magy, Pauline Chazelas, Laurence Richard, Nathalie Deschamps, Simon Frachet, Jean-Michel Vallat, Corinne Magdelaine, Frédéric Favreau, Flavien Bessaguet, Anne-Sophie Lia, Mathilde Duchesne

**Affiliations:** 1 UR20218—NEURIT, Limoges University, 87000 Limoges, France; 2 Service de Neurologie—CHU Limoges, 87000 Limoges, France; 3 Service de Biochimie et Génétique Moléculaire—CHU Limoges, 87000 Limoges, France; 4 Service de Bioinformatique—CHU Limoges, 87000 Limoges, France; 5 Service d’Anatomie Pathologique—CHU Limoges, 87000 Limoges, France

**Keywords:** early diagnosis, CANVAS, sensory neuropathy, chronic cough

## Abstract

CANVAS, a rare disorder responsible for late-onset ataxia of autosomal recessive inheritance, can be misdiagnosed. We investigated a series of eight patients with sensory neuropathy and/or an unexplained cough, who appeared to suffer from CANVAS, and we emphasized the clinical clues for early diagnosis. Investigations included clinical and routine laboratory analyses, skin biopsy, nerve biopsy and molecular genetics. The eight patients had clinical and/or laboratory evidence of sensory neuronopathy. All but one had neuropathic pain that had started in an asymmetric fashion in two patients. A chronic cough was a prominent feature in our eight patients and had started years before neuropathic symptoms in all but one. The course of the disease was slow, and ataxia remained mild in all. Five patients were initially thought to have immune-mediated sensory neuronopathy and received immunotherapy. Skin biopsies showed a near complete and non-length-dependent loss of intraepidermal nerve fibers. Moreover, nerve biopsy findings suggested a prominent involvement of small myelinated and unmyelinated fibers. The burden of CANVAS extends far beyond cerebellar ataxia and vestibular manifestations. Indeed, our study shows that a chronic cough and neuropathic pain may represent a major source of impairment in these patients and should not be overlooked to allow an early diagnosis and prevent unnecessary immunotherapy.

## 1. Introduction

Cerebellar ataxia, neuropathy, vestibular areflexia syndrome (CANVAS) is a rare disorder of autosomal recessive inheritance. The recently identified mutations of the replication factor C subunit 1 (*RFC1*) gene allow us to better delineate the phenotypic spectrum of this condition [1]. In 2019, Cortese et al. identified a biallelic intronic AAGGG repeat expansion between exons 2 and 3 in the RFC1 gene as the molecular cause of CANVAS. In the controls, both motifs (such as AAAAG, AAAGG and AAGAG) and the size of the nucleotide repeats (usually 11 repeats or more) differ from the pathologic one (Cortese et al., 2019; Akçimen et al., 2019). The RFC1 gene, located on chromosome 4 (4p.14), contains 25 exons and encodes for the large subunit of replicator factor C (RFC) which is a clamp loader involved in the DNA replication fork. It occurs in adulthood, and an electrodiagnostic study identifies sensory neuronopathy as a key feature of this disorder [2]. As mixed ataxia of sensory and cerebellar/vestibular origin is reported to be the main cause of disability in CANVAS [3], the involvement of large sensory myelinated fibers has been emphasized, but pinprick sensation decrease in many patients suggests there is, indeed, an involvement of small myelinated fibers and/or of unmyelinated fibers as well [4]. In addition, in a recent large series of patients with genetically proven CANVAS, neuropathic pain was present in 42 and symptoms of autonomic involvement in 32 of 105 patients, respectively [5]. Moreover, an unexplained cough is a frequent and often early feature in CANVAS, pointing to a possible involvement of the small fibers of the sensory neural pathways regulating cough [6]. Here, we investigated a series of eight patients from six unrelated families, in whom we confirmed a CANVAS diagnosis using molecular biology. We present here the clinico-pathological findings and emphasize the particularities of a chronic cough, neuropathic pain, and skin and nerve biopsy in these patients.

## 2. Materials and Methods

This study was conducted in accordance with the Declaration of Helsinki and was approved by our local ethics committee under the number 448-2021-104. All patients described in this paper gave their signed, informed consent for use of their clinical and paraclinical data.

### 2.1. Patients

Five out of the eight patients included in this study were referred to our department for sensory symptoms suggesting sensory neuropathy/neuronopathy. Two patients were investigated because they are first-degree relatives of patients diagnosed with CANVAS, and only one other patient was referred for the initial suspicion of this disorder because of a chronic unexplained cough.

### 2.2. Genetic Testing

Blood samples were collected in EDTA tubes after providing informed consent. The protocol was in accordance with French ethics legislation and the Declaration of Helsinki. Genomic DNA was extracted by standard methods (Illustra DNA Extraction kit BACC3, GEHC Amersham UK). Polymerase chain reaction (PCR) flanking the repeat was performed using the recently described protocol with slight modifications [1]. Repeat-primed PCRs (RP-PCR) for the nonpathogenic AAAGG and AAAAG and for the pathogenic AAGGG repeat expansions in RFC1 were performed as described by Cortese et al. [1] and the RP-PCR for the AAGAG repeat expansion as recently described [7].

### 2.3. Other Laboratory Testing

Nerve conduction studies were performed on our eight patients during routine diagnostic testing using standard methods [8]. In addition, blood samples were obtained from 5 of our patients in whom the diagnosis of CANVAS was not initially suspected, mainly to rule out alternative diagnoses of sensory neuropathy and included: viral serodiagnosis for Hepatitis B, C and HIV; Borrelia serodiagnosis; blood vitamin levels and immunologic testing to search for Sjögren’s disease; cryoglobulinemia; antiganglioside antibodies; monoclonal gammopathy; and paraneoplastic syndromes. CSF examination was performed in four patients. Four patients underwent brain MRIs to look for cerebellar atrophy, and two patients underwent conventional vestibular testing.

### 2.4. Skin Biopsy

After informed consent, seven patients (all except patient 8) had skin biopsies at the proximal thigh and distal leg, and samples were processed as described [9]. Intraepidermal nerve fiber density (IEFND) was manually counted under a NIKON DxM1200 light microscope. The density was calculated in, at least, three nonconsecutive sections as the number of IENF per millimeter as described earlier [10].

### 2.5. Nerve Biopsy

After informed consent, four patients (1, 3, 6, 7) underwent sural or radial (patient 1) nerve biopsy, respectively. Samples were processed as described earlier [11]. Specimens were fixed in 2.5% glutaraldehyde. After dehydration processing, tissues were embedded in Epon. Semi-thin sections (1 mm) were stained with toluidine blue, and ultrathin sections (90 nm) were stained with lead citrate and uranyl acetate then observed under an electron microscope (JEOL JEM-1400 Flash, Tokyo, Japan). Photographs were slightly modified only for white balance, exposure and contrast, and figures were assembled using Microsoft PowerPoint.

## 3. Results

### 3.1. Main Clinical Findings

There were eight female patients with a mean age at referral of 57.4 +/− 4 years (range 52–62). The cause of referral was sensory neuropathy of an unexplained origin in five patients (one, three, five, six, seven). Patients two and four were sisters of patients one and three, respectively, and patient eight was referred because of a chronic unexplained cough, which had prompted a nerve conduction study leading to a diagnosis of sensory neuronopathy (see Table 1).

Sensory symptoms had been present at referral in all but one patient (patient eight) for 4.9 +/− 2 years (range 3–8). When present, they were generally symmetrical with involvement of the distal legs at the beginning and with a chronic course, except in patient three, where it started with painful sensations of the lateral aspect of the right leg and in patient five, where it started with painful sensations of the left foot and in the left trigeminal nerve territory. Along with numbness, patients with sensory symptoms had painful sensations that were described as sharp stabbing pain, pins and needles, and electric shock sensations mainly involving the legs, sometimes in a random fashion. Painful sensations tended to exacerbate in the evening or at night.

Clinical examination showed no or mild ataxia in six out of the eight patients (see Table 1) and at a normal strength in all. Tendon jerks were normal in all but three patients (one, six, seven) who had absent reflexes of the Achilles. Sensory examination showed extensive sensory impairment (at least in a stocking-glove distribution) for superficial and deep sensations in all patients. Loss of vibration sense was particularly prominent in all patients and up to the collar bone for patient three. Additionally, all patients (including patient eight who had no sensory complaint) had decreased pinprick sensation, at least, in the four extremities. In addition, all patients, except patient eight, complained of mild swallowing difficulties.

At the time of referral, all but one patient (patient seven), had an unexplained cough that had lasted for 16 +/− 4 years (range 15–20). All these patients had a persistent irritating dry cough, which was triggered by a variety of factors such as emotion, stress, speaking, ear cleaning with a cotton stalk or swallowing. The cough was never worse at night or in a supine position, ruling out swallowing difficulties as its main cause. Otherwise, it had a relentless evolution with no seasonal fluctuations. All patients with a cough had visited many different practitioners including pneumologists, gastroenterologists and otorhinolaryngologists. The cough was generally thought to be caused by an allergy or gastroesophageal reflux (although it was not influenced by positional changes in our patients). Interestingly, patients one and five were treated with 60 mg extended release codeine (Dicodin LP^®^) at night, which led to an almost complete disappearance of cough. Patient eight had been using antihistaminic drugs for more than 10 years with modest efficacy.

### 3.2. Initial Diagnosis and Course

Five patients (one, three, five, six, seven) had been followed for five to ten years. None of these patients had an initial diagnosis of CANVAS. For patients one, three and seven, the occurrence of attacks of sudden neuropathic pain was considered typical of an acquired cause. Moreover, patients one and three had abnormalities at work-up that were suggestive of Sjögren’s disease and paraneoplastic sensory neuronopathy, respectively. Patient five had trigeminal neuralgia, which was also considered suggestive of an acquired origin. All these five patients, who were initially considered to have immune-mediated sensory neuronopathy received intravenous immunoglobulin or oral steroids. Patients six and seven who received IVIg had a subjective improvement of pain and balance. None of these patients had initially mentioned their chronic cough. In these five patients, the progression of sensory symptoms was very slow, with almost no worsening. Similarly, ataxia had remained mild at the last evaluation, with all patients being able to walk unaided. Indeed, all patients in this series described neuropathic pain and/or a chronic cough as their most disabling symptoms.

### 3.3. Laboratory Investigations

All patients had normal routine blood tests, and no one had autoantibodies (e.g., antinuclear, anti-SSA/SSB or antiglycolipid antibodies). CSF examination was performed in four patients and was always normal. Patient one had a grade 3 focus score at her minor salivary gland biopsy that led to the initial erroneous diagnosis of Sjögren’s disease. Patient three, who had an asymmetric onset of sensory symptoms had a positive PET scan suggesting a suspicious lesion at the duodenal level. This led to suspecting a paraneoplastic sensory neuronopathy, but onconeural antibodies were absent and endoscopic examination ruled out a neoplastic lesion. Four patients underwent brain MRIs, which showed a mild cerebellar atrophy in all cases (see Table 1). Two patients (three and five) had undergone complete vestibular testing because of occasional dizziness, showing bilateral vestibular hyporeflexia.

### 3.4. Molecular Analysis

For all patients, no product could be obtained by flanking PCR suggesting the presence of large expansions. In addition, a saw-tooth pattern on the RP-PCR was obtained for the pathogenic AAGGG repeat expansion for all these patients, associated to negative RP-PCR for the three other conformations. The molecular diagnosis of CANVAS was then confirmed for these eight patients.

### 3.5. Skin and Nerve Biopsy Findings

#### 3.5.1. Skin Biopsy

The seven patients who underwent skin biopsy had an almost complete loss of intraepidermal nerve fibers (and an important decrease in subepidermal nerve plexuses as well) at the distal leg and proximal thigh (see Table 1). Notably, loss of IEFN was as important at the thigh as at the leg, which suggested a non-length-dependent axonal degeneration in favor of a sensory neuronopathy [12,13].

#### 3.5.2. Nerve Biopsy

**Figure 1:**
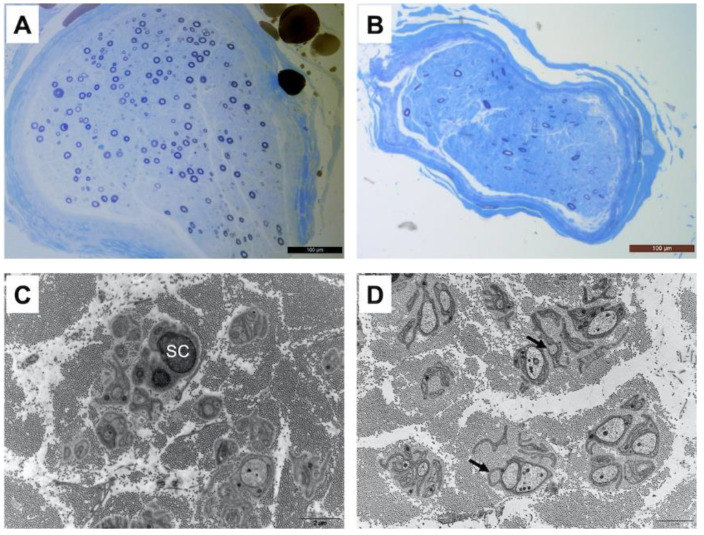
Moderate to severe loss of large and small diameter myelinated fibers in the radial nerve of patient 1 (**A**) and in the sural nerve of patient 3 (**B**) Resin-embedded semi-thin transverse sections stained with toluidine blue. On electron microscopic examination, small fibers around a Schwann cell (SC) are largely disorganized with several extensions and occasional stacks of membranes in the radial nerve of patient 1 (**C**). The same pattern is observed in the sural nerve of patient 3 (**D**) with the presence of dispersed collagen pockets (arrows). Transverse ultrathin sections of radial and sural nerves stained with uranyl acetate and observed under an electron microscope (scale bar 2 μm for (**C**,**D**)).

Patient one underwent a radial nerve biopsy, whereas patients three, six and seven had a sural nerve biopsy. In all patients, interstitial tissue examination after paraffin embedding and hematein–eosin staining did not show any inflammatory infiltrate or abnormal deposits. Semi-thin sections obtained from epon-embedded tissue and stained with toluidine blue showed an important loss of large and small diameter myelinated fibers, without any sign of ongoing regeneration (no clusters) as shown in Figure 1. The loss of myelinated fibers was homogeneous between the different fascicules in each observed sample. Electron microscopic examination confirmed the loss of small myelinated fibers. Additionally, there were obvious abnormalities of unmyelinated fibers with many abnormal extensions and stacks of basal membranes occasionally forming collagen pockets (Figure 1). It should provide a concise and precise description of the experimental results, their interpretation, as well as the experimental conclusions that can be drawn.

## 4. Discussion

Cerebellar ataxia, neuropathy, vestibular areflexia syndrome (CANVAS) is a rare autosomal recessive disorder of adulthood, with core clinical features which are sensory loss caused by sensory neuronopathy, progressive ataxia with cerebellar involvement and signs of vestibular dysfunction [5]. It is caused by a biallelic expansion of an intronic AAGGG repeat in the replication factor C subunit 1 (RFC1) gene [1]. Late-onset ataxia is a common presentation of this rare disorder, and the value of systematic vestibular testing has been largely emphasized [14]. In addition, a sensory neuronopathy is a constant finding in CANVAS with sensory signs seeming to involve all type of fibers, but little has been written on sensory symptoms in this disorder [4]. Taking into account the recent literature, CANVAS should be now considered in the differential diagnosis of any sensory neuronopathy and/or ataxia, as well as paraneoplastic and immune-mediated sensory neuropathies, mitochondrial disorders and hereditary ataxias including Friedreich’s disease.

Two of the eight patients reported here had sensory symptoms that were asymmetrical at the beginning, and one patient had trigeminal neuralgia. Positive sensory symptoms (e.g., paresthesia, pins and needles or neuropathic pain) are seldom encountered in hereditary neuropathy, and their occurrence in an asymmetrical manner in adulthood would usually point to an acquired disorder. Indeed, most of our patients had been initially considered to have an immune-mediated sensory ataxic neuronopathy, which prompted us to look for Sjögren’s disease, paraneoplastic sensory neuronopathy or ganglionopathy linked to anti-FGFR3 antibodies [15]. However, as recently emphasized, the partial or complete sparing of tendon reflexes in our patients is a typical feature of CANVAS [16,17]. Therefore, it must be kept in mind that sensory neuronopathy with positive sensory symptoms, even with an initial asymmetrical distribution, might be encountered in CANVAS.

We found in all our patients who underwent skin biopsy an extremely severe and non-length-dependent loss of intraepidermal nerve fibers, a feature that has been previously described in two patients with genetically unconfirmed CANVAS [18]. However, the involvement of small fibers in CANVAS has been largely neglected. Indeed, in a recently published large series of patients with this rare disorder, it was stated that ‘patients with biallelic RFC1 expansions do not exhibit insensitivity to pain, with painless injuries, ulcerations and amputations, suggesting that A-delta and C fiber sensory neurons are selectively spared in this disorder’ [5]. On the contrary, our findings demonstrate in our patients a near complete loss of IEFN at the skin level and an important loss of small myelinated and unmyelinated fibers in the sural or radial nerve for the four patients that underwent a nerve biopsy. Indeed, small fiber involvement and neuropathic pain had been emphasized in patients with CANVAS with pathological evidence of unmyelinated fiber injury [19].

The involvement of small fibers in CANVAS is probably clinically important. Indeed, one concern in seven of our patients, who had no or negligible ataxia, was the presence of neuropathic pain. Clinical signs of small fiber impairment in CANVAS had been previously reported, and this feature has to be taken into account in these patients [4]. As recently outlined, various sensory symptoms including neuropathic pain due to the involvement of small fibers might be important for the quality of life of patients who suffer small fiber injury [20].

In addition, all our patients had a chronic spasmodic cough of an unexplained origin, which preceded the sensory symptoms by years in all but one case. The cough was disabling in most patients, with attacks triggered by various stimuli. Notably, the cough was not exacerbated at night or in a supine position, suggesting that it was not caused by swallowing disorders or by gastroesophageal reflux. Patients with this rare disorder are said to have an unexplained cough, often preceding the other signs of the disease by decades [5,21]. A chronic cough is a complex disorder, which may be linked to pulmonary or extrapulmonary conditions. In some instances, it can be considered a neuropathic disorder, where the cough is triggered by low-level stimuli not normally sufficient to cause a cough and by smaller amounts of known cough-inducing stimuli [22]. It may be linked to the denervation of the upper airways and esophagus and is often associated with gastroesophageal reflux [23]. Moreover, cough hypersensitivity syndrome (CHS) is related to the sensitization of sensory nerves in the epithelium of airways that originate from neurons derived from the vagal nerve [6]. Interestingly, these neurons are divided into two groups: the chemosensitive nociceptors in jugular ganglia (unmyelinated C-fibers) and the low-threshold mechanosensors in nodose ganglia (myelinated A-delta fibers) which are activated by touch-like mechanical stimuli [24]. Whether the cough in CANVAS is due to injury of the peripheral afferent pathways of the vagal sensory ganglia with sensitization and denervation of the upper airways and esophagus is highly probable but remains to be elucidated [6,19]. Nevertheless, from our findings indicating that small fibers are involved in CANVAS, one may speculate that, indeed, C and/or A-delta fiber pathology may be implicated in the sometimes severe and intractable cough of these patients [22]. Considering a cough as a possible neuropathic symptom in patients with CANVAS, it would seem logical to use the same type of drugs that are used on neuropathic pain such as antiepileptic drugs (gabapentine, pregabaline, lacosamide or carbamazepine) or antidepressants (tricyclic antidepressants or duloxetine). Opioids could also represent an alternative in patients who do not respond to these drugs.

Due to the distribution of sensory symptoms and the occurrence of neuropathic pain, five of our patients were initially considered to have a possible immune-mediated sensory neuronopathy, and in two of them, results of laboratory investigations erroneously seemed to confirm this hypothesis. This led to the use of immunomodulatory treatments that were ineffective and could have been harmful. This emphasizes the need to suspect CANVAS in patients with sensory neuronopathy, even with positive and asymmetric sensory symptoms.

## 5. Conclusions

Finally, the clinical picture of CANVAS involves sensory ataxia and vestibular disturbances, but neuropathic pain and a chronic cough may be very disabling for patients with this disorder. Although a chronic unexplained cough is a classical feature of this disorder, it is not always mentioned by patients seen by neurologists because they do not think it may be linked to their neuropathic symptoms. However, the clinical and electrodiagnostic findings in our patient eight demonstrates that even in the presence of an isolated chronic cough, without any sensory symptoms, widespread involvement of the peripheral nervous system might be present. Therefore, physicians should be aware that a complete neurological evaluation is useful in patients who present with an unexplained dry spasmodic cough.

## Figures and Tables

**Table 1:** Main clinical and laboratory features at referral of eight female patients with CANVAS.

Patient	Age at Referral	Family History	Duration of Sensory Symptoms (Years)	Initial Suspected Diagnosis	Pain	Duration of Unexplained Cough before Neurological Presentation (Years)	Ataxia at Referral	Cerebellar Atrophy	NCS Pattern	IEFND at the Thigh/Leg (Fibers/mm)
1	62	Yes	5	Immune-mediated	Yes	20	No	ND	SNN	0.25/0.49
2	57	Yes	5	CANVAS	Yes	10	Mild	ND	SNN	0/0.53
3	52	Yes	3	Immune-mediated	Yes	10	Mild	Slight	SNN	0/0
4	52	Yes	3	CANVAS	Yes	20	No	Slight	SNN	0/0
5	61	No	7	Immune-mediated	Yes	20	Mild	Slight	SNN	0.34/0
6	58	No	3	Immune-mediated	Yes	15	Moderate	ND	SNN	0/0.17
7	62	No	8	Immune-mediated	Yes	NA	Moderate	Slight	SNN	0.51/0.37
8	55	No	NA	CANVAS	No	15	Mild	ND	SNN	ND

IEFND: intraepidermal nerve fiber density; NCS: nerve conduction study; NA: not applicable; ND: not done; SNN: sensory neuronopathy. Normal values for IEFND for our lab are >9 at the thigh and >6 at the leg.

## Data Availability

The data supporting reported results can be obtained on demand.
